# Image-Processing Scheme to Detect Superficial Fungal Infections of the Skin

**DOI:** 10.1155/2015/851014

**Published:** 2015-11-16

**Authors:** Ulf Mäder, Niko Quiskamp, Sören Wildenhain, Thomas Schmidts, Peter Mayser, Frank Runkel, Martin Fiebich

**Affiliations:** ^1^Institute of Medical Physics and Radiation Protection, Technische Hochschule Mittelhessen - University of Applied Sciences, 35390 Giessen, Germany; ^2^Helmut Hund GmbH, Artur Herzog Straße 2, 35580 Wetzlar, Germany; ^3^Institute of Bioprocess Engineering and Pharmaceutical Technology, Technische Hochschule Mittelhessen - University of Applied Sciences, 35390 Giessen, Germany; ^4^Department of Dermatology, Venereology and Allergology, Justus Liebig University Giessen, 35390 Giessen, Germany

## Abstract

The incidence of superficial fungal infections is assumed to be 20 to 25% of the global human population. Fluorescence microscopy of extracted skin samples is frequently used for a swift assessment of infections. To support the dermatologist, an image-analysis scheme has been developed that evaluates digital microscopic images to detect fungal hyphae. The aim of the study was to increase diagnostic quality and to shorten the time-to-diagnosis. The analysis, consisting of preprocessing, segmentation, parameterization, and classification of identified structures, was performed on digital microscopic images. A test dataset of hyphae and false-positive objects was created to evaluate the algorithm. Additionally, the performance for real clinical images was investigated using 415 images. The results show that the sensitivity for hyphae is 94% and 89% for singular and clustered hyphae, respectively. The mean exclusion rate is 91% for the false-positive objects. The sensitivity for clinical images was 83% and the specificity was 79%. Although the performance is lower for the clinical images than for the test dataset, a reliable and fast diagnosis can be achieved since it is not crucial to detect every hypha to conclude that a sample consisting of several images is infected. The proposed analysis therefore enables a high diagnostic quality and a fast sample assessment to be achieved.

## 1. Introduction

It is assumed that 20 to 25% of the global human population is affected by superficial fungal infections, with a constantly increasing incidence [1]. In tropical areas they are a major cause of morbidity due to the ideal warm and humid conditions for fungal growth [2]. The dermatophytes, a major cause for the infections [3, 4], digest keratin and can therefore be found on skin and its annexes (hair, nail) [5]. They are transmitted through direct person-to-person contact or indirectly through desquamated infected epidermis or hairs [5].

Due to the widespread occurrence and the resulting large number of patients, it is a frequent task for dermatologists to diagnose and to treat fungal infections. Direct microscopic examination is generally used as a screening method, because it is fast and cost-effective [6]. Fluorescence staining increases sample contrast and therefore further facilitates the detection of fungi [7, 8]. A drawback of microscopy is that no information on the fungal species can be obtained. Hence, additional methods such as fungal culture or DNA-based polymerase chain reaction methods have to be performed, whenever information about the fungal species is important [9]. However, direct microscopic examination is considered an essential method for the diagnosis of superficial fungal infections [6].

Although microscopy is faster and cheaper than culture- or DNA-based methods, it has some drawbacks. Depending on user experience, sample condition, and sample size, it may still be time-consuming to evaluate complete samples. Diagnosing multiple samples at once may therefore be a tedious task that could lead to classification errors and increased intra- and interobserver variability.

To overcome these drawbacks, an image-analysis scheme has been developed that automatically detects fungal infections in digital fluorescence microscopy images. The use of image-processing methods to detect fungal structures is a common approach in biotechnology for the characterization and analysis of fungal growth in fermentation processes [10–13]. However, the automated evaluation of clinical images of fungal infections is, to our knowledge, a new topic.

The developed analysis scheme should be useful for clinical routine and has to be designed to meet the specific requirements. Most importantly, in addition to a high sensitivity and specificity, a reliable diagnosis should be available during patient contact time. Hence, the image analysis and the visualization of the results have to be adapted to the clinical workflow. In this context it is necessary to reduce the time-to-diagnosis to as great an extent as possible. This can be accomplished by choosing algorithms with low calculation time and by online visualization of the detection results.

The approach presented in this study used multiple image-processing steps to preprocess, segment, and parameterize the images taken with an automated fluorescence imaging system. The parameters to describe the detected structures were used in a rule-based classification scheme to decide whether a fungal infection is present. Image-processing methods were chosen to achieve acceptable calculation times. The method's performance was evaluated for manually chosen test datasets and clinical images of infected and uninfected patients.

## 2. Materials and Methods

### 2.1. Sample Material and Preparation

Infected samples consisting of small skin scales were taken from clinical cases in a university hospital. The samples were gathered during routine examination where fungal infections were diagnosed by clinicians. Additionally, uninfected samples from healthy subjects were also taken at the hospital. The sample preparation consisting of maceration and staining with commercially available MykoColor (RSC Pharma, Giessen, Germany) was performed at our laboratory. The skin scales were located on an object slide and 0.02 mL of MykoColor was added. A cover glass was used to gently flatten the sample.

### 2.2. Imaging Device

The imaging was performed using an experimental automated fluorescence imaging system (Helmut Hund GmbH, Wetzlar, Germany) that provided a complete 1 cm^2^ area scan (consisting of 100 single images) of the object slide on which the skin scales were randomly located. The system is equipped with a monochrome camera (5 megapixel), an objective (10x magnification, 0.25 NA), and an LED illumination unit (excitation peak: 365 nm) and is capable of autofocusing on sample structures.

### 2.3. Description of Images, Hyphae, and Other Structures

**Figure 1:**
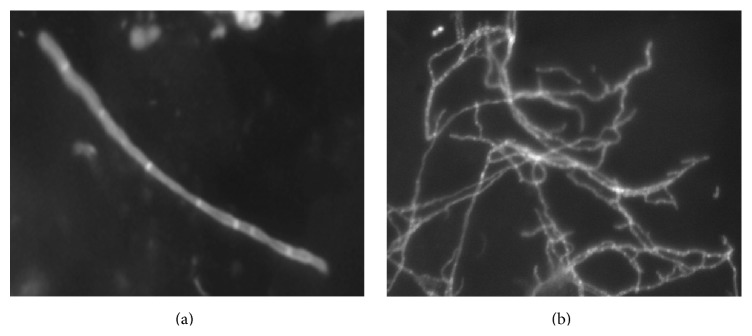
Image of a singular (a) and clustered (b) hyphae using the automated fluorescence imaging system.

The fluorescence images of the samples captured with the automated device typically show a dark background with bright fluorescent structures. These structures are either hyphae that belong to a fungal infection or false-positive structures and artefacts that can be misinterpreted as hyphae.  Figure 1 shows the two classes of hyphae, singular (a) and clustered (b), which can be generally observed and which were under investigation in this study. Singular hyphae are characterized by relatively uniform width and intensity. The shape is often elongated or curved without branches. The clustered hyphae can be described as an agglomeration of overlapping singular and branched hyphae that may have multiple junctions.

False-positive structures that were present in the samples were mainly cellulose fibers of clothing, circular and irregular reflections of the illumination unit occurring at air inclusions, and other miscellaneous objects such as plastic particles and dirt.

**Figure 2:**
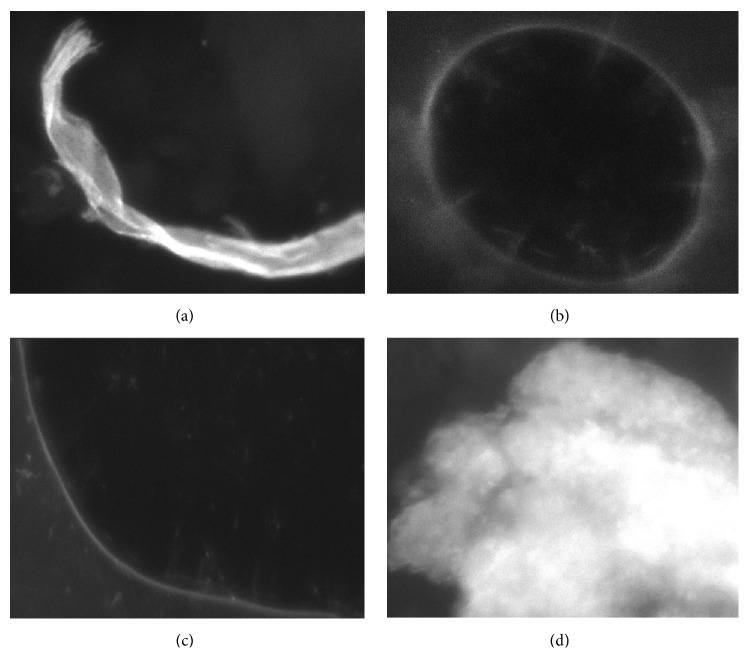
False-positive structures: cellulose fiber (a), circular reflection (b), irregular reflection (c), and miscellaneous structures such as dirt or plastic particle (d).

Cellulose fibers (see Figure 2(a)) can often be found in skin samples. The characteristic features are the elongated but irregular surface and texture with varying widths. Furthermore, the fibers are often larger than hyphae.

Circular and irregular air inclusions (Figures 2(b) and 2(c)) occur during the sample preparation process. Small inclusions form circular structures with bright transitions between air and the staining reagent due to reflections of the illumination unit. Larger inclusions show various irregular shapes with similar transitions as small inclusions.

Miscellaneous structures are external contaminations such as plastic particles from the sample containers (Figure 2(d)) used for transport and storage, dirt, and dust. The shape of miscellaneous objects is variable but in most cases different than hyphae. Additionally, these objects can be identified due to the high intensities.

**Figure 3:**
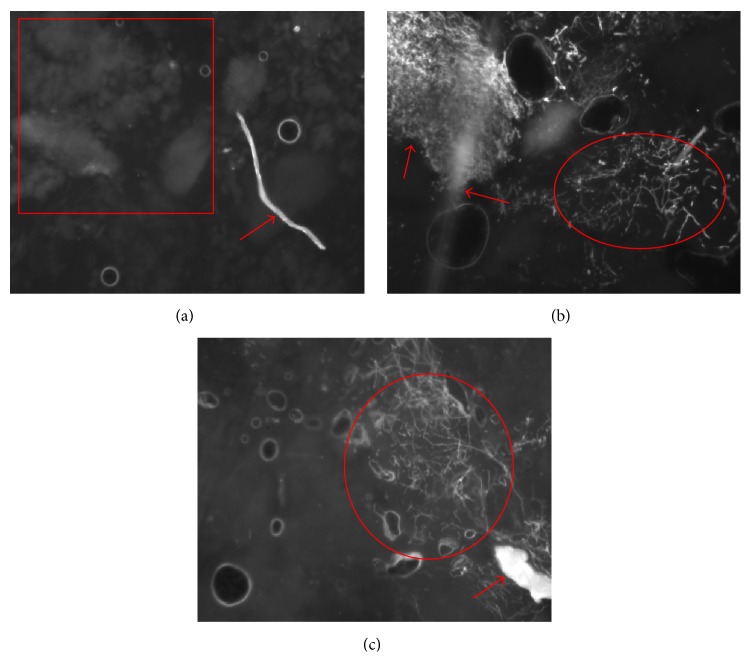
Exemplary overview of clinical images. Fungal infection is indicated by elliptical markers (b, c). The rectangular marker (a) represents the extracted skin scales. False-positive structures are indicated by arrows: cellulose fiber (a), obscuring and misc. particles (b, c). Circular and irregular air inclusions are present in all images.

Figure 3 shows an exemplary overview of the clinical images with multiple structures like skin scales, cellulose fibers, and miscellaneous particles that can often be found. The images indicate the variety of suspicious objects that have to be dealt with while detecting hyphae and the challenges in evaluating the data for dermatologists and software algorithms.

### 2.4. Image Analysis

**Figure 4:**
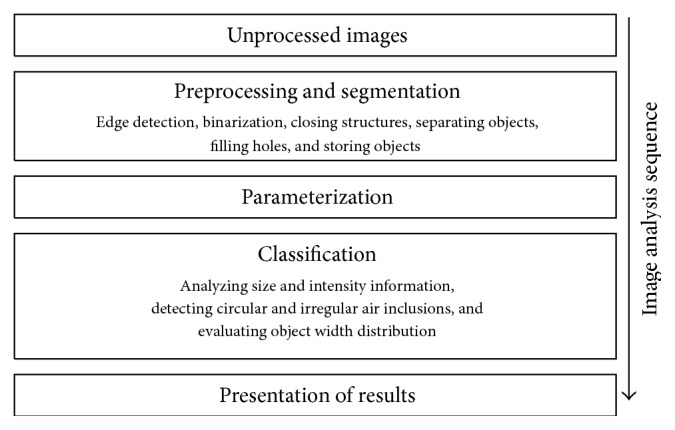
Overview of the analysis scheme.

The developed image-analysis scheme (overview shown in Figure 4) is divided into the stages of image preprocessing and segmentation, parameterization, and object classification. The open-source image-processing framework OpenCV [14] is used for the implementation. Furthermore, a graphical user interface was developed to load image data and to visualize the results.

Preprocessing and segmentation starts with the Canny-Algorithm [15] for background reduction and detecting structure edges. After binarization, the algorithm closes discontinuous objects (using morphological dilatation) and extracts connected structures into single region of interests (ROI) using the connected components approach based on a region growing algorithm.

For filling holes, which might be present in the objects, the next preprocessing step extends the ROI by a one-pixel wide border to separate objects from the ROI margin. Then a flood filling algorithm is used to assign an arbitrary intensity value to all pixels surrounding the object. In the last step we assign the object intensity value to all pixels that do not correspond to the arbitrary intensity value.

After preprocessing all single objects are stored in a dataset that is used in the successive parameterization and classification steps.

**Figure 5:**
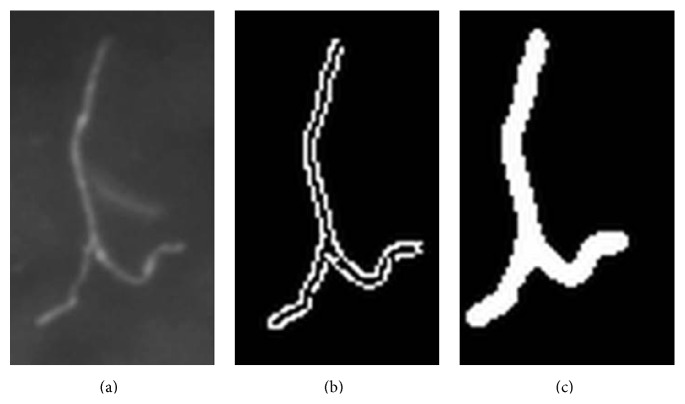
Image preprocessing performed on a hypha. (a) Original image detail. (b) After segmentation using Canny-Algorithm. (c) After closing and filling of holes.

Figure 5 shows the result of each preprocessing step for a hypha.

In the parameterization step morphological and statistical features are calculated for every suspicious object in the dataset. It has to be mentioned that all thresholds stated below are only valid for the used imaging system and camera setup. Hence, they ought to be reviewed on other systems. First, a preselection of the objects by object size and object intensity is performed.

*Object Size*. To sort out small artefacts that are often present in the samples, only objects that consist of a certain amount of pixels are considered in the classification. For the used imaging setup a threshold of 250 pixels was manually chosen based on the size distribution of hyphae.

*Object Intensity.* The exposure time and the gain of the used camera are configured to match the brightness of stained hyphae. Therefore, a threshold for the mean intensity of the structures (value of 130 for the used setup) was investigated to sort out very bright false-positive structures. 

Then, the following features are calculated for the remaining suspicious objects. A subsequent rule-based classification uses these features and thresholds to exclude false-positive objects (no hyphae) from the dataset of objects and to keep true-positive hyphae. 

*Histogram Analysis*. The intensity distribution of irregular air inclusions shows a characteristic first peak at low intensities and a second peak on the falling slope of the first peak. This is due to the fact that air and staining reagent yield different intensity values in the image. Therefore, ROIs containing irregular air inclusions contain two background intensity levels which are only present in air inclusions. This information is used to distinguish these structures from hyphae. For the identification of the characteristic shape the position and the amplitude of the peaks in the smoothed histogram of the objects are calculated and compared to manually derived thresholds (position of peak 1 not after bin 30 and amplitude of peak 2 not lower than 15). These thresholds were obtained by evaluating all observed irregular air inclusions. 

*Detecting Circular Structures*. An algorithm based on the Hough Transformation principle [16] for circular structures is used to exclude circular air inclusions. As the surrounding rectangle of circular objects is quadratic, the algorithm is calculated only for objects with a width-to-height ratio between 0.8 and 1.2. The pixel intensities along a circle centered at the middle of the surrounding rectangle with the corresponding radius are summed up and normalized to the circles perimeter. A threshold of 10% of the pixels lying on the circle was investigated to decide that the object is a circular air inclusion. 

**Figure 6:**
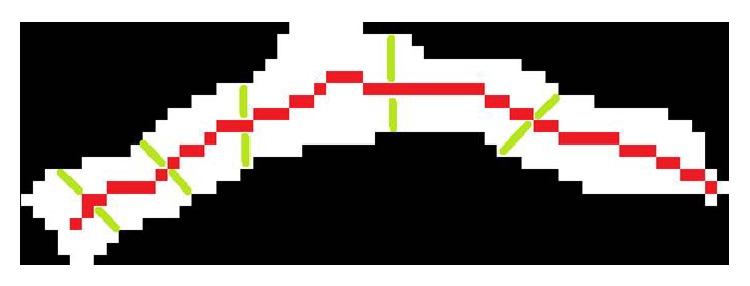
Binarized representation of a singular hypha (white pixel) with skeleton (red line) and perpendicular width evaluation along the exemplarily shown green lines.

*Width Analysis*. As hyphae are of uniform width and show a smooth surface, the width distribution perpendicular to the structures skeleton is calculated between two adjacent pixels for every other pixel position (example shown in Figure 6). Furthermore, junctions in the skeleton are detected to distinguish between singular and clustered hyphae. The mean thickness and the standard deviation are subsequently compared to thresholds (for singular hyphae: mean thickness between 4.4 and 9 pixel, standard deviation lower than 2.8; for clustered hyphae: mean thickness between 4.4 and 10 pixel, standard deviation lower than 12). Using this information, irregular structures such as cellulose fibers, plastic particles, other contaminations, and too thin or thick structures such as air inclusions are identified and excluded from the dataset of infected structures. 

After the classification all identified false-positive objects are excluded from the dataset and only true-positive hyphae are left. For presenting the results the images in which infected objects are found are visualized in the graphical user interface.

### 2.5. Specification of Classification Parameters

A test dataset consisting of subsets of manually selected objects of all occurring true- and false-positive findings was created to specify the classification features. 100 singular hyphae, 70 clustered hyphae, 90 circular reflections, 90 irregular reflections, 44 cellulose fibers, and 19 miscellaneous particles were chosen for the subsets. The classification parameters shown above were manually optimized in an iterative process to yield the highest sensitivity. The classification results for the test dataset are shown in Tables 1 and 2, respectively. As only objects of sufficient size were chosen, the performance of the “object size” criteria was not evaluated.

### 2.6. Evaluation

The overall performance of the method for clinical fluorescence microscopy images using the automated imaging system was evaluated. Therefore, the sensitivity, specificity, and calculation time for a total of 415 images were investigated. These images were initially classified into “infected” and “uninfected” by experienced clinicians. The algorithm was used for the automated classification and the results were compared.

The results are presented on a “per image” perspective, which means that a given image is recognized as infected as soon as one hypha is found. Hence, the “per image” perspective can be used to reach a correct classification without detecting all hyphae that are present in the image. The correct classification of an uninfected image, by contrast, means that the algorithm has to sort out every false-positive structure.

The “per image” evaluation method is chosen because of the clinical relevance of single images. Dermatologists do not need to recognize all hyphae that are actually present in a sample. Theoretically, it is sufficient to detect one hypha to diagnose a fungal infection.

## 3. Results

All calculations were performed on an Intel Core 2 Quad Q9650 CPU at 3.00 GHz. Although the image import and the image processing are performed separately on single cores, no parallelization of the image processing itself has been implemented so far.

### 3.1. Performance of Classification

The results of the classification for the test dataset of true- and false-positive structures are shown in Table 1. In the given context, “classified correctly” means that objects are recognized as hyphae for true-positives and that objects are not recognized as hyphae for false-positives.

The detection rate after optimizing the classification parameters of the algorithm is 94% and 89% for singular and clustered hyphae, respectively. Regarding the false-positive structures the detection rate for circular reflections is 98%, for irregular reflections 86%, for cellulose fibers 86%, and for miscellaneous particles 89%.

The performance of the classification features on false-positive structures is shown in Table 2. The detection rate varies for the different types (circular reflection, irregular reflections, cellulose fibers, and misc. particles) of false-positive structures. The “object intensity” feature was especially useful for cellulose fibers and miscellaneous particles. The circularity measure and histogram analysis sorted out false-positives due to reflections of air inclusions successfully and the width calculation detected false-positives of all classes.

Furthermore, the Canny-Algorithm used for segmentation has a strong influence on the classification performance. In the case of circular reflections the algorithm sorted out 50 false-positive objects due to their low intensities. On the other hand, the algorithm did not detect all originally continuous objects as singular objects. It tends to split large structures into multiple objects. This effect can be observed for cellulose fibers and miscellaneous particles.

A more detailed evaluation of the algorithm showed that the methods used to reduce false-positive structures wrongly excluded true-positive hyphae from the dataset of suspicious objects. The width calculation, the detection of circular structures, and the histogram analysis each sorted out two singular hyphae. For clustered hyphae five and three objects were excluded by the width calculation and the histogram analysis, respectively (data not shown).

### 3.2. Performance on Clinical Images

The developed algorithm detected 160 out of 194 infected images and 174 out of 221 uninfected images correctly. Hence, the total sensitivity is 83% and specificity is 79% for clinical fluorescence microscopy images (see Table 3). In total, 8,433 objects were segmented using the background reduction. After rejecting small structures, 2,311 objects remained for classification.

Analysis of the processing steps showed that the calculation times per object and per image were 18 and 96 milliseconds for preprocessing, 18 and 101 milliseconds for segmentation, and 11 and 61 milliseconds for parameterization including classification, respectively. The average calculation time for the whole processing was 47 milliseconds for an object and 258 milliseconds for an image (see Table 4).

## 4. Discussion

An image-analysis scheme to detect fungal infections in digital fluorescence microscopy images is presented. The scheme consists of the image preprocessing, segmentation, parameterization, and classification steps that all have a strong influence on the detection rate. Results show that the classification parameters yield high detection rates for hyphae (94% and 89% for singular and clustered hyphae, resp.) of the test dataset. However, the overall performance on clinical images is lower. This can be explained by the different image quality of the test dataset and the clinical images. As the aim of the test dataset was the evaluation of the algorithm's sensitivity for morphological and statistical features, the only objects that were chosen are those of high image quality in terms of sharp contours and high object-to-background contrast. The image quality of the clinical images, on the other hand, is poor compared to the test dataset. The object-to-background contrast can be low because other structures overlap the hyphae. Additionally, due to the sample thickness, some structures may be located in out-of-focus areas and therefore appear blurred.

However, the “per image” approach increases the sensitivity as it is sufficient to detect one hypha to diagnose an infection. In this context, it has to be considered that, as a consequence of fungal growth, often multiple singular and clustered hyphae are present in infected samples. The likelihood for correctly classifying images as infected therefore increases with an increasing number of hyphae.

The same results can be observed for the specificity of the method. The detection rate of false-positive structures of the test dataset is higher than the overall specificity. Here, the “per image” approach deteriorates the results as an image often consists of multiple false-positive structures that all have to be classified correctly. In particular irregular air inclusions, cellulose fibers, and miscellaneous particles can reduce specificity, since more than 10% of the objects are classified incorrectly (see Table 2).

Therefore, the high sensitivity's tradeoff is the lower specificity, which, in consequence, is responsible for a high number of uninfected objects that are detected as hyphae. To facilitate the process of diagnosis for the dermatologists images classified as true-positive are presented without highlighting the detected objects. In this way well experienced dermatologists can decide more quickly whether an image contains hyphae as they do not explicitly need to evaluate every single object.

The limitation of the current evaluation is that the analysis is performed on a “per image” base and not on a “per patient” base. The “per image”-approach is used to optimize the processing algorithms, because it is more sensitive for slight improvements of the algorithms. Using this approach a more profound insight into the performance is gained and optimization potentials can be derived. However, to assess the system's performance on a clinical trial for numerous subjects the “per patient” approach has to be used and a ROC analysis [17] has to be performed.

The analysis of the calculation times shows that an image is completely classified within an average time of 258 ms. This means an infection can be diagnosed directly after starting the processing if it is present within the first images. On the other hand if no infection is present or the infection is located in the last images, it takes about 30 seconds (assuming that 100 images are scanned per sample) to receive the complete results.

Minimizing calculation time has the main drawback that complex segmentation and classification algorithms that are computationally expensive cannot be implemented in this context. The segmenting of elongated objects such as hyphae could be performed, especially, by active contour models as presented by Liu et al. [18]. The drawback of this iterative method is that the segmentation time for an easy cone-like object on an ideal image background is about one second and up to about three seconds on a noisy background. For elongated vessels that resemble fungal hyphae, calculation time is stated to be about 13 to 141 seconds depending on the iterations used and the desired segmentation quality. Inglis and Gray also report expensive processing time for contour segmentation for clustered hyphae [19]. These algorithms provide very accurate outlines of the hyphae but are too slow to be used in the context of clinical routine diagnosis. To use these complex algorithms in future works, calculations will have to be speeded up. Parallelization or using GPU calculations might be a promising approach.

However, the less complex edge-detection approach for segmentation used in this study has also been reported to successfully detect hyphae or elongated structures in images. Baum et al. describe a processing framework called HyphArea that uses an edge-detection algorithm based on the Sobel operator [20] to measure fungal growth in biotechnological applications. Kumar et al. report the successful use of the Canny edge-detection filter to find vessel contours in cross section images [21]. Our study also showed good segmentation results; due to various inhomogeneities of image quality and interfering objects, however, some structures are separated into multiple objects.

In comparison to studies performed for measuring biomass and characterizing growth processes of fungi in fermentation processes, the processing schemes used are similar to the approach presented here. Papagianni reviewed commonly used approaches in biotechnology and summarizes the outline of the image-processing steps as follows: image enhancement, image segmentation, object detection, binary image processing, measurements/calculations, and data analysis [13]. This outline is also implemented in the presented study.

The results of the reviewed studies showed very good segmentation performance [10–12, 22, 23]. The most important difference of clinical dermatological images used in our study compared to biotechnology images is the increased complexity and wide variability of structures. Biotechnology approaches do not need to deal with false-positive structures, unintended contaminations, and reduced perceptibility of structures due to varying sample conditions, meaning that segmentation results cannot be readily compared.

In general, the parameters of the preprocessing and classification algorithms themselves need to be configured very thoroughly. All processing steps rely on size and intensity information of the images. They depend on the magnification used, the numerical aperture of the objective, the illumination unit, and the camera. Therefore, the values and thresholds used in this study are valid only for the used experimental imaging system and are shown as examples. Considering that the morphology of fungal hyphae and false-positive objects does not vary with changing imaging systems the presented parameterization approach can generally be transferred. For the classification, in contrast, it is unlikely that images from other systems, taken under different conditions, can be evaluated successfully without tuning the thresholds, unless image resolution, magnification, image quality, and fluorescence intensity are preserved. In this study, however, the algorithm was exclusively developed for the presented imaging system and no further assessment on the needed effort to transfer the results to other imaging platforms was performed.

Furthermore, it has to be considered that the results depend on the performance of the algorithms but can be optimized by careful sample preparation. Reducing the incorporation of false-positive structures during transportation and storage and trying to avoid air inclusions during staining are important steps to increase specificity and to minimize the time needed for diagnosis. To increase sensitivity, it is useful to assure uniform sample thickness to avoid out-of-focus structures in the images. This can be achieved by ensuring an adequate time for the maceration and application of the staining reagent and applying sufficient pressure to the sample.

The benefits and drawbacks of supporting medical personnel with computed “second opinions” have been discussed in the field of radiology [24] since the early 1990s. Here, the so-called computer-aided diagnosis (CAD) is nowadays a common and widely accepted tool to improve the accuracy and consistency of radiological diagnosis and also to reduce the image reading time [25, 26]. Gurcan et al. report that the approaches have also been transferred to pathological images, an area in which CAD algorithms have begun to be developed for disease detection, diagnosis, and prognosis prediction to complement the opinion of the pathologist. Gurcan et al. further describe the need for quantitative image-based assessment to complement the educated but subjective opinion of pathologists [27]. The approach presented in this study follows these ideas and transfers the CAD methodologies to the dermatological field of superficial fungal infections.

## 5. Conclusion

The presented method can speed up the process for the diagnosis of fungal infections for dermatologists. It provides standardized and reproducible results that help to increase overall diagnostic quality in this field. To be applicable in clinical routine, the microscope, probe preparation, and software have to be combined into a commercially available automated imaging system.

## Figures and Tables

**Table 1:** Classification results for the test dataset for true- and false-positive structures.

	True-positive structures		False-positive structures			
	Hyphae: singular	Hyphae: cluster	Circular reflection	Irregular reflection	Cellulose fiber	Misc. particles
Total number	100	70	90	90	44	19
Classified correctly	94	62	88	77	38	17

Detection rate	94%	89%	98%	86%	86%	89%

**Table 2:** Performance of the processing steps in the reduction of false-positive structures.

	Circular reflections	Irregular reflections	Cellulose fibers	Misc. particles
Total number in test dataset	90	90	44	19
After segmentation	40	90	47	30
Sorted out by				
Intensity	0	0	17	12
Circularity	21	0	0	2
Histogram analysis	2	65	1	0
Width calculation	15	12	23	14

Recognized as hyphae	2	13	6	2
Detection rate	98%	86%	86%	89%

**Table 3:** Total performance of the algorithm for 415 clinical fluorescence microscopy images.

	Infected images	Uninfected images
Total amount	194	221
Classified correctly	160	174

Classified correctly in %	83	79

**Table 4:** Calculation time per object and per image for preprocessing, segmentation, and parameterization including classification based on the 415 clinical images. Data are rounded.

Calculation time	Preprocessing	Segmentation	Parameterization	In total
Per object [ms]	18	18	11	47
Per image [ms]	96	101	61	258

In total for 415 images [s]	40.0	41.7	25.2	106.9
